# Mental Health Conditions Among E-Learning Students During the COVID-19 Pandemic

**DOI:** 10.3389/fpubh.2022.871934

**Published:** 2022-05-17

**Authors:** Anna Rutkowska, Błazej Cieślik, Agata Tomaszczyk, Joanna Szczepańska-Gieracha

**Affiliations:** ^1^Department of Physical Education and Physiotherapy, Opole University of Technology, Opole, Poland; ^2^Department of Kinesiology and Health Prevention, Jan Dlugosz University in Częstochowa, Częstochowa, Poland; ^3^Descartes' Error Student Research Association, Faculty of Physical Education and Physiotherapy, Opole University of Technology, Opole, Poland; ^4^Faculty of Physiotherapy, University School of Physical Education, Wroclaw, Poland

**Keywords:** COVID-19, mental health, depression, anxiety, e-learning, Poland

## Abstract

**Aim:**

The COVID-19 pandemic has forced the education system to undergo changes, which have also affected universities. E-learning became the main form of education, reducing interpersonal contacts, which could affect the mental wellbeing of students. The aim of this study was to investigate the prevalence of depressive symptoms and the level of perceived stress during e-learning among Polish students and to identify the factors for predicting higher levels of depression symptoms.

**Methods:**

The study included 753 participants with a mean age of 22.47 (±4.02) years. The Perception of Stress Questionnaire (PSQ) and Beck Depression Inventory (BDI-II) were used to measure the severity of stress and level of depression. Furthermore, our own survey was used to assess the impact of e-learning on various aspects of life. To examine how much stress can explain a statistically significant amount of variance in depression, three-step hierarchical multiple regression was used. In addition, our own questionnaire was used to assess the impact of e-learning on education, social contacts and technical abilities.

**Results:**

A total of 58% of the students characterized by an increased level of stress. 56% show symptoms of depression and 18% of the participants had suicidal thoughts. The most significant predictor of depression is high stress levels and factors related to e-learning: isolation from friends and acquaintances, negative impact on level of knowledge, reduced motivation to learn, and worsening grades. This predictors may explain about 66% of the variance of depression.

**Conclusion:**

Universities should implement interventions and educational programmes, providing ad *hoc* assistance in the form of individual or group meetings with a psychologist (also in a remote form) and organizing workshops and webinars on strategies for managing stress.

## Introduction

In March 2020, the World Health Organization declared that the Coronavirus Disease 2019 (COVID-19) was a pandemic. Since then, with the increase in infections, the social situation of every citizen of the world has changed (1, 2). From March 20, 2020, according to the decree of the Minister of Health, COVID-19 has been an epidemic in Poland. Temporary physical isolation steps have been implemented to stop the spread of the virus and prevent the health care system from being overloaded. Since March 12, all educational institutions have been closed. School children and university students have been pursuing remote learning for many months. This situation at universities continued until 1 October 2020. Thereafter, depending on the pandemic situation and the restrictions introduced, education was provided in a combined form. This has disrupted the regular pattern of education and standard practices that have been followed for many years. It has been a great organizational challenge for the academics but most importantly for the students (3, 4).

Remote learning has resulted in long-term social isolation and limitations in interaction with peers. This situation may lead to an increased level of loneliness among young people. A literature review of 63 studies involving 51 576 participants found a clear association between loneliness and mental health problems in children and young people in particular with the prevalence of depression (5). Although we still do not know the long-term global consequences of this situation, research suggests that the pandemic has caused serious damage to public mental health (6, 7). The public has lost a sense of security, which triggered strong emotional reactions, causing feelings such as anger, fear, irritability, frustration, increased stress, insomnia, nervousness, anxiety, and depressive symptoms (8–11). According to the authors' analysis in reviews of literature, the prevalence of stress and depression in the general population as a result of the pandemic is 29 % and 33 % respectively (12) and 36% of stress and 28% of depression (13). It has also been observed that about 30% of students have symptoms of depression and 50% increased levels of perceived stress, during the COVID-19 pandemic (14, 15). The studies presented show that students are in a group exposed to higher levels of perceived stress.

While the Internet and social networks allow young people to communicate, socialize, and learn during a pandemic, they also come with negative consequences. Research indicates that spending excessive time on social media correlates with increased levels of depression, anxiety, and psychological distress (16). There is also a significant decrease in physical activity levels among students as a result of social isolation, which may be another factor that affects mental health (17–20).

It is important to note that a high risk of anxiety, depression, and chronic stress has been observed among young people even before the pandemic outbreak, and this trend has been observed in studies for a number of years (21–24). As shown in an international meta-analysis, the prevalence of depression among pre-pandemic students worldwide was 30.6% (21). This number is much higher than the prevalence of depression of 12.9% in the global population before pandemic (25). In Poland, between 1998 and 2020, the perceived level of depression among Polish students ranged from 6% to 30% (26–31). The literature reports that there is a correlation between depressive symptoms and the prevalence of suicidal thoughts and suicide among young adults. The literature reports that adolescent survivors of suicide attempts were later diagnosed with depression (32, 33).

In published reports, researchers warn that psychological interventions and the inclusion of psychiatric care in future crisis management plans should be an integral part of pandemic health management (34–36). Therefore, it is important to implement research that will provide an up-to-date picture of the state of this problem. This will allow social attention to be diverted toward the needs of young people and the development of prevention and support programmes.

Due to the dynamic nature of the pandemic, it is important to monitor the mental state of young people, especially in the context of ongoing e-learning. Therefore, it was decided to investigate the prevalence of depressive disorders and the level of perceived stress during ongoing remote learning in a group of students from the Polish University, as well as to identify the impact of e-learning on various aspects of life. The following research hypotheses were formulated: (1) E-learning and the resulting barrier to interpersonal relationships among students increased perceived stress levels and the risk of depression; (2) increased levels of stress in students have an impact on depressive symptoms.

## Methods

### Participants

A total of 753 students from Opole University of Technology, representing 17.64% of all students, with an average age of 22.47 (±4.02) participated in the survey. Students represented all university departments at various levels of higher education. The survey was conducted between January 11 and 17, 2021 during the remote learning period, which has been near-continuous since March 12, 2020. In-person teaching was resumed at the Opole University of Technology on 1 October, 2020 and then suspended by the decision of the rector. Since October 12, e-learning has been implemented in all fields of study. The study was conducted using an online survey that included standardized psychological questionnaires; the Perception of Stress Questionnaire (PSQ) and the Beck Depression Inventory (BDI), a self-administered questionnaire with responses on a 5-point Likert scale, and demographic questions. The invitation to participate in the study with a link to the survey form was sent to all 4,267 students from the Opole University of Technology through the e-learning platform (Moodle).

The study was approved by the Research Ethics Committee of the University of Physical Education in Wrocław, Poland (Ref. No. 1/2021) and the study was registered in ClinicalTrials.gov (NCT04926441). Each participant gave their voluntary consent to participate in the study, which was fully anonymous.

### Perception of Stress Questionnaire (PSQ)

The PSQ measures generalized stress and allows for a more accurate assessment of stress in three dimensions: emotional stress, intrapsychic stress, and external stress. The questionnaire contains 27 statements that the respondent addresses by selecting responses on a 5-point Likert scale (true, rather true, hard to say, rather not true, or untrue). The higher the score obtained on a scale, the higher the level of stress experienced.

Interpreting the overall score between 21 and 105, with a cut-off point of 60, allows the determination of the participant's stress level. Raw scores were also converted to a STEN (standard ten) scale. STEN 1–2 indicates a very low score, STEN 3–4 indicates a low score, STEN 5–6 indicates an average score, STEN 7–8 indicates a high score, and STEN 9–10 indicates a very high score. The questionnaire obtained reliability indicators at the level of: external stress: α = 0.72, emotional tension: α = 0.81 and intrapsychic stress: α = 0.69 (37). In our study, Cronbach's α for the total stress level was 0.93, external stress: α = 0.79, emotional tension: α = 0.81, and intrapsychic stress: α = 0.81.

### Beck Depression Inventory (BDI)

BDI is a tool used to determine the severity of depressive symptoms. The 21-item questionnaire consists of two parts: emotional and somatic. Depending on the number of points obtained, the severity of depression can be assessed. A score of 0–10 indicates that there is no depression, 11–27 indicates moderate mood disorder, and 28 or above indicates major depressive disorder. The cutoff for categorizing participants into depressive and non-depressive subgroups was a score of 10, following the guidelines given by Beck et al. (38). In addition, individual questions concern, inter alia, fear for the future, suicidal thoughts, or sleep problems. In the group of people with an increased level of depression, the answers to the questions indicated in the text were analyzed and the percentage of people was determined on this basis. In our study, Cronbach's α was 0.92.

### The Experience of E-learning During a Pandemic

Participants also completed a self-administered questionnaire containing 8 questions. The survey contains two domains on the impact of e-learning on various aspects of life. The survey responses were structured on a 5-point Likert Scale: (1) Strongly disagree; (2) Disagree; (3) Neither agree nor disagree; (4) Agree; (5) Definitely agree. The questions were grouped into two areas: distance learning and technical domain. The domains were extracted using factor analysis (varimax-rotated principal components analysis)(Supplementary Table S1). The questionnaire's content was discussed with the students of the scientific association “Descartes' Error” at the Opole University of Technology. The questionnaire showed satisfactory reliability with a Cronbach alpha of 0.82.

Distance learning Domain/Questions

1. E-learning isolated me from my friends.
2. E-leaning has had a negative impact on my level of knowledge.
3. E-learning has had a negative impact on my level of practical skills.
4. E-learning reduced my motivation to learn.
5. I think I will get poorer grades on exams due to distance learning.

Technical Domain/Questions:

1. Due to the pandemic, my skills in using modern technologies have improved.
2. E-learning forced me to upgrade my computer (purchase of new hardware or software).
3. I use a PC which satisfies my needs.

### Statistical Analysis

Data were analyzed using SPSS (Version 24.0, SPSS Inc., Chicago, IL). Demographic characteristics, depression, and stress level are presented with means, standard deviations (SD), and percentages. The percentages of responses to other questions were calculated according to the number of respondents per response and the number of total responses to a question and presented as categorical variables. In all analyzes, the female gender was coded as 1 and the male as 0. The associations between depression, stress level, and stress domains were evaluated with Pearson's r correlation. Multiple regression (hierarchical) was used to determine which variables of psychological characteristics predicted the prevalence of depression and stress symptoms. As independent variables, gender, age, social, technical and stress domains were used. The α level was set at 0.05 for establishing statistical significance.

## Results

### Participant Characteristics

A total of 753 participants completed the survey, all of whom were included in the final sample. The mean age of the respondents was 22.47 (±4.02) years and approximately half of them were women (48.37%). Most of the respondents were first-year students (30.01%). About 10% had a chronic illness and 14% took medication regularly. Table 1 presents the characteristics of the study participants.

**Table 1:** Participants demographics.

**Variables**	**Total group** ***n*** **= 753**				
	***M***	**SD**	**Range**	***n***	**%**
Age years	22.47	4.02	17-54		
Gender	48.37				
Women				388	51.53
Men				365	48.47
Faculty					
Physical Education and Physiotherapy				211	28.02
Mechanical Engineering				38	5.05
Civil Engineering and Architecture				87	11.55
Economics and Management				102	13.55
Production Engineering and Logistics				80	10.62
Electrical Engineering, Automatic Control and Computer Science				235	31.21
Year of study					
First				226	30.01
Second				168	22.31
Third				165	21.91
Fourth				134	17.80
Fifth				60	7.97
Study type					
Full-time				618	82.07
Part-time				135	17.93
Economic deterioration during lockdown			1–5	334	44.36
Presence diagnosis of chronic diseases				76	10.09
BDI	14.01	10.12	0–47		
0–10				328	43.55
11–27				341	45.29
28 and more				84	11.16
General stress level	65.03	18.30	21–102		
Emotional tension	24.54	7.27	7–35		
External stress	19.92	5.94	7–35		
Internal stress	20.57	6.84	7–35		

*M, mean; SD, standard deviation; BDI, Beck Depression Inventory*.

### Authors' Survey, Depression Symptoms, and Stress Level Results

More than half of the respondents agreed with the statement that e-learning isolated them from friends and acquaintances (59.9%) and that it had a negative impact on their level of knowledge (50.4%). Similar results were obtained in the questions concerning the impact of e-learning on the reduction of motivation to learn (58.6%) and the deterioration of their obtained grades (60.1%).

The mean BDI score was 14.01 (±10.12), 45.3% were above the first cut-off point and had mild symptoms of depression, and 11.1% had moderate or severe symptoms of depression. A total of the 56% of students show symptoms of the depression and 18% of participants had experienced suicidal thoughts. Taking into account the distribution of responses with elevated depression symptoms (BDI >10), 94.6% of the students revealed fear for the future, 90.4% of them had feelings of sadness and depression. A similar proportion experienced nervousness and irritability (86.8), difficulty getting motivated (83.3%), difficulty making decisions (82.8), and stated that they fell self-dissatisfaction (82.8%).

**Figure 1:**
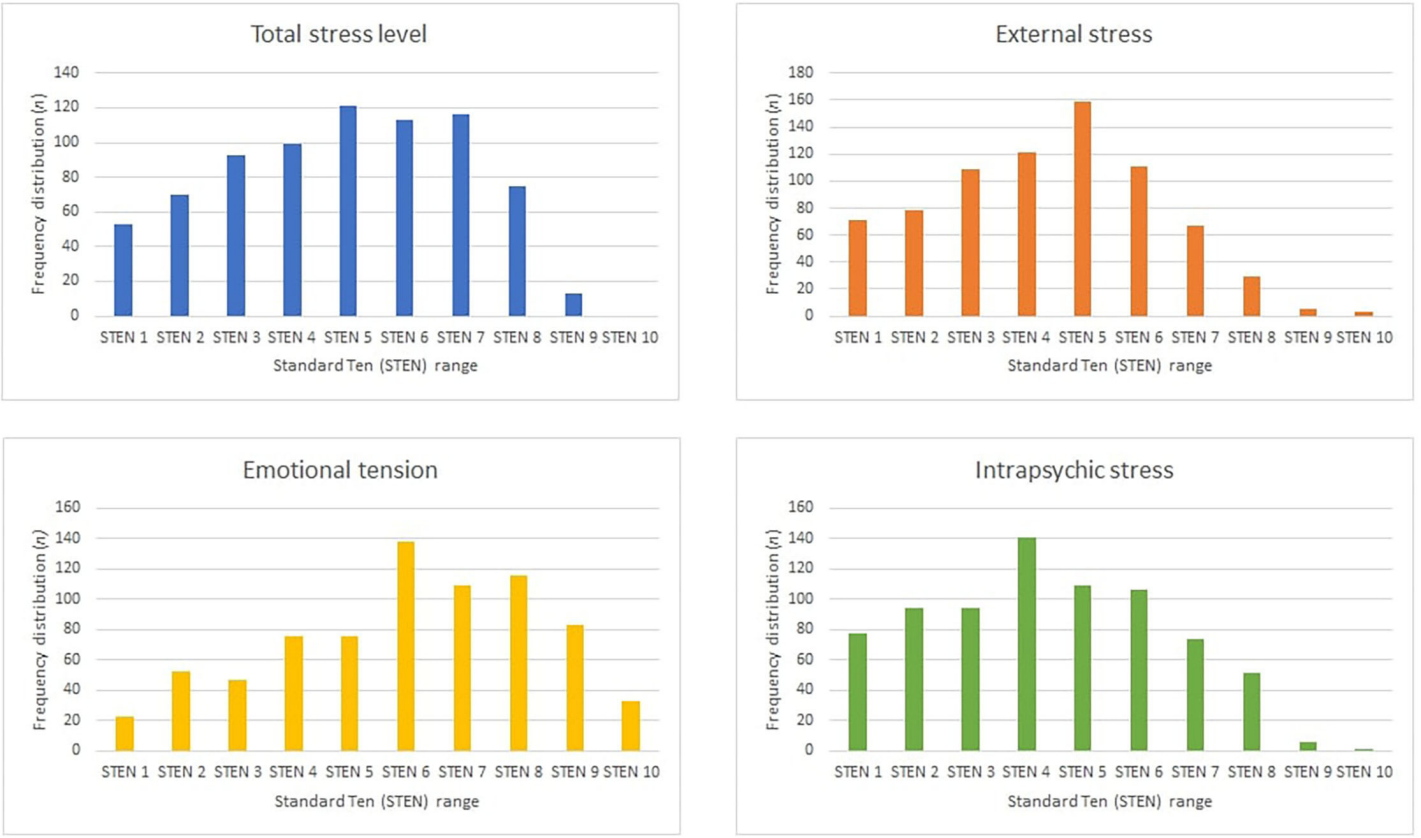
Distribution of the respondents in the respective STENs.

Figure 1 illustrates the distribution of the respondents in the respective STENs. Among the participants, the raw general stress level was 65.03 (±18.30) out of 105, External tension = 24.54 (±7.27), External stress = 19.92 (±5.94) and Intrapsychic stress = 20.57 (±6.84). Considering the STEN results regarding the general stress level, 31.08% of people were in the 5–6 range and 27.09% in the 7–10 range. In terms of emotional tension, 28.42% of the participants were in the range 5 to 6 and 45.29% in the 7–10 range; for external stress, 35.86% were in the range 5 to 6 and 13.81% in the range 7–10; and for internal stress, 53.92% were in the range 5 to 6 and 17.53% were in the range 7–10.

**Figure 2:**
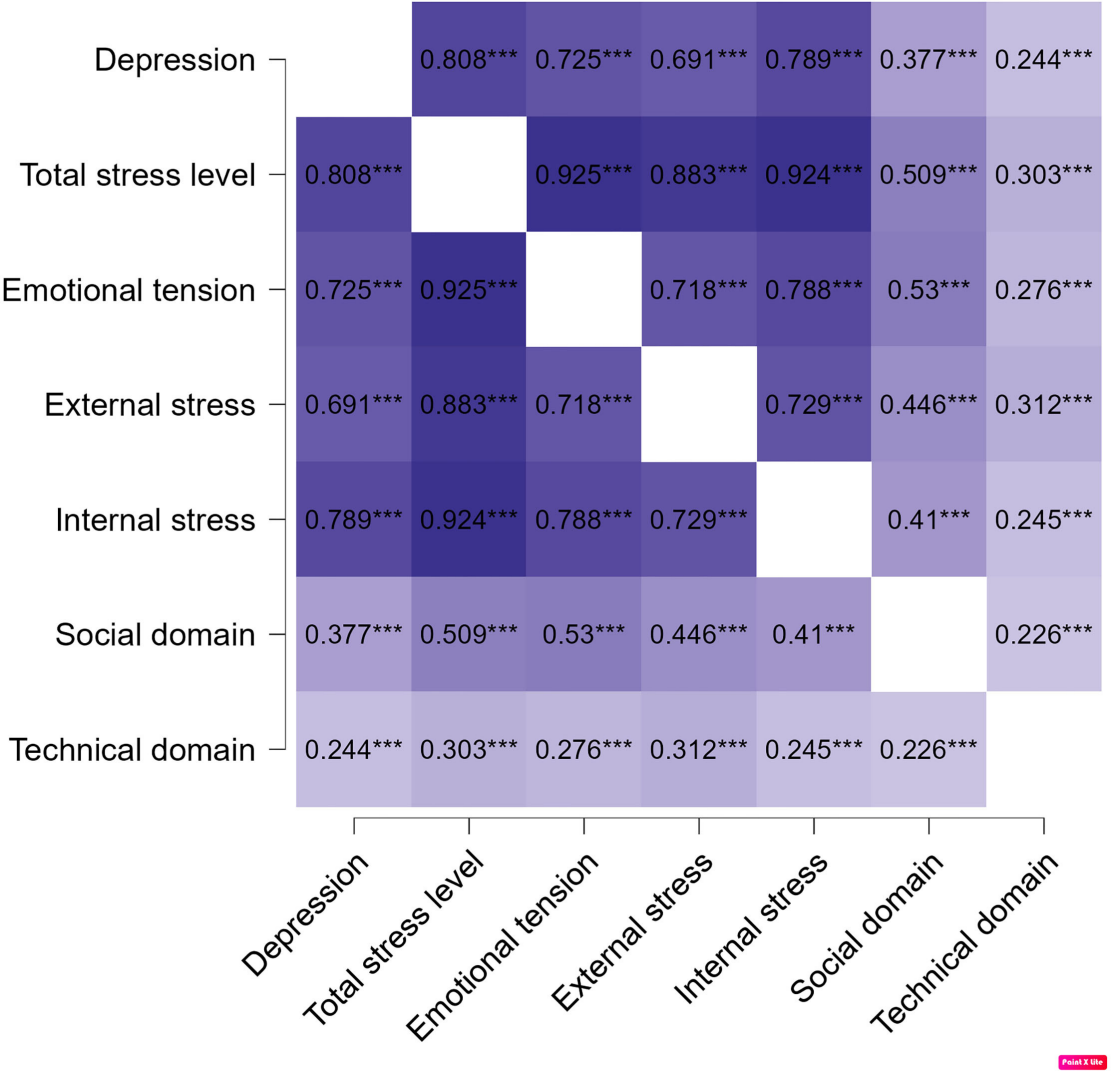
Correlation heatmap for depression, stress level and stress domains.

In Figure 2 presents correlation heatmap for depression, stress level and stress domains. All parameters demonstrated a strong positive correlation (*p* < 0.01). Pearson's correlation coefficient ranged from 0.24 to 0.93 (Figure 2).

### Factors for Predicting Higher Levels of Depression Symptoms

In order to examine how much stress can explain a statistically significant amount of variance in depression, three-step hierarchical multiple regression was used. In the first step of the regression model, the gender and age variables were included and were found to be an important predictor variable, accounting for 2% of the variance of depression (*p* = 0.02)(Table 2). In the second step, distance learning and technical domains were added to the regression model. Among the variables included in the second model, only the gender did not reveal significant predictors. This model accounted for 18% of the variance in depression. As shown in Table 2, in the third step, the interaction effect between age, gender, distance learning, technical, and stress domains was examined. In the third regression model, the interaction between stress domains was found to be a significant predictor. The third model may explain about 66% of the variance of depression (*p* < 0.001).

**Table 2:** Results of stepwise regression for depression.

**Variables**	**b**	**SE b**	**95% CI**		**t**	***p***	**F**	**df**	***p***	**R^**2**^**	**ΔR^**2**^**
			**LL**	**UL**							
**Step 1**							6.63	2	0.001	0.02	0.02
Gender	1.80	0.73	0.36	3.24	2.46	0.01					
Age	−0.24	0.09	−0.42	−0.06	−2.63	0.01					
**Step 2**							39.81	4	<0.001	0.18	0.16^**^
Gender	0.91	0.68	−0.41	2.24	1.35	0.18					
Age	−0.18	0.08	−0.34	−0.01	−2.11	0.03					
Distance learning domain	0.549	0.058	0.44	0.66	9.46	<0.001					
Technical domain	0.782	0.157	0.47	1.09	4.97	<0.001					
**Step 3**							212.03	7	<0.001	0.66	0.49^**^
Gender	0.10	0.43	−0.75	0.95	0.24	0.81					
Age	−0.06	0.05	−0.16	0.05	−1.07	0.28					
Distance learning domain	−0.04	0.04	−0.13	0.04	−1.05	0.29					
Technical domain	0.06	0.10	−0.14	0.27	0.63	0.53					
Emotional tension	0.30	0.05	0.19	0.41	5.56	<0.001					
External stress	0.31	0.06	0.20	0.43	5.48	<0.001					
Internal stess	0.724	0.06	0.62	0.83	13.10	<0.001					

** *p < 0.01. COVID-19, Corona Virus Disease 2019; PSQ, Perception of Stress Questionnaire; BDI-II, Beck Depression Inventory; SD, standard deviations*.

## Discussion

This self-reported study evaluated the effect of distance learning on perceived stress and depressive symptoms and identified the factors with the strongest effects on mental health among university students. The obtained results indicate that many months of e-learning and social isolation negatively affect the mental state of young students, causing symptoms of depression and stress. More than half of the respondents reported feeling sad and depressed, nervousness and irritability, and decreased social contact. Students also indicate a decrease in motivation to learn and believe that e-learning had a negative impact on their level of knowledge and will receive worse grades.

A total of the 56.4% of students experienced depressive symptoms. Taking into account the distribution of responses with elevated depression symptoms (BDI >10), 94.6% of the students revealed fear for the future, 90.4% of them had feelings of sadness and depression. A similar proportion experienced nervousness and irritability (86.8), difficulty getting motivated (83.3%), difficulty making decisions (82.8), and stated that they fell self-dissatisfaction (82.8%).

Scientific reports are available from many countries on the prevalence of depression among students during a COVID-19 pandemic. A comprehensive systematic with meta-analysis presents the most up-to-date and extensive estimate of the prevalence of college students' depressive symptoms by collecting data from 89 observational studies covering a total of 1,441,828 students. The overall combined prevalence of depressive symptoms during the COVID-19 pandemic was determined to be 34% (39). For example, in Liban, a total of 33.4% (*n* = 520) of students reported depressive symptoms; in China: 48.3 (*n* = 4,872); in Spain: 31.19% (*n* = 2,530); in France: 49.5% (*n* = 69,054); and in the USA: 48.14% (*n* = 2,031) (40–44). A study of students in Greece showed similar results to our study, in which it was shown that depressive symptoms affected up to 60.9% (*n* = 1,000) of the participants (45). Researchers have attempted to identify factors that affect mental health during a pandemic. They are probably due to the quality of health services, cultural, political, and economic differences (13) Single young adults in particular are more vulnerable to mental health disorders (46). The student population, even before the pandemic, has been mentioned as being at risk of poor mental health relative to other groups (7, 21, 22, 47).

It is alarming that 18% of the students in this study reported suicidal thoughts. This is significantly higher than the results published in previous years, where the average was 10% (47–49). High values were also observed among French students (11.4%), American students (18.04%) and Greek students (20.2%) during the pandemic (43–45). Researchers report that a major risk factor for suicide in this age group is the prevalence of depression (50). In addition, the risk of suicide doubles during the university study compared to after graduation (51).

Our study used three-step hierarchical multiple regression to identify predictors of depression prevalence. We found that factors related to e-learning could significantly influence approximately 18% of the variance in depression. University students represent a population that is particularly vulnerable to mental health problems, in light of the challenges associated with their transition into adulthood, independence, and often facing economic and material difficulties (52). It seems that numerous problems and negative emotions were increased with prolonged e-learning. Students in the self-study indicated that e-learning decreases their motivation to learn (58.6%), has a negative impact on their level of knowledge (50%), and will cause deterioration of their grades (60,1%). More than half of the students agreed with the statement that e-learning isolated them from friends and acquaintances (59.9%). Alongside knowledge acquisition, the development of interpersonal relationships is a priority during the university years. The friendships and partnerships formed during this time often last for the rest of students' lives.

However, the biggest predictor of depression is high stress levels. The correlation between stress and depression has long been documented in the scientific literature. Studies in young adults also suggest that stress is a predictor of depression (53–55).

In our study, subjects are also characterized by an increased level of stress (58.17%). PSQ allowed identification of the most significant factor causing stress; emotional stress (45.29%) is the most commonly experienced by students, characterized by excessive irritability and nervousness, feelings of anxiety, inability to relax, fatigue, and lack of desire and energy to act. This study was conducted 10 months after the announcement of the pandemic and the introduction of remote learning. A similar study was conducted at Opole University of Technology in March 2020 during the global lockdown. The researchers estimated that elevated stress levels were present in a total of 56% of the subjects (56). Therefore, it can be concluded that prolonged e-learning and apparent adaptation to the situation do not reduce stress levels, suggesting that students have not implemented effective strategies to reduce stress levels. In published research on stress levels among students during the covid pandemic, the values ranged from 24.7 to 71.2% (43, 56–61). It is significant to note that before the pandemic, stress levels were assessed mainly in students in stress-prone fields such as medicine, nursing and dentistry, whereas now the focus is on students in all disciplines.

As the number of COVID-19 cases continues to increase steadily, the pandemic-related mental health disorders that exist will continue to affect millions of people around the world. Understanding how different populations are affected would provide a basis for identifying those at high risk. Attention should be directed toward the development of new e-learning approaches as a holistic educational concept, rather than just a collection of information to be learned. A possible solution for improving students' mental health might be to offer breaks during classes for a few minutes of physical activity or relaxation training. University teachers should participate in workshops and training that provide modern tools for remote operation, aimed at increasing motivation for learning and appropriate verification of knowledge and its reflection in the form of grades. According to the findings, it seems very important to foster telemental health projects that offer remote support to individuals suffering from mental health problems during lockdown.

Although the sample size in the study is large and representative of technical universities, the results of this study cannot be generalized to the entire student population in Poland or other countries. Further research is required to compare the wellbeing of students around the world. It remains to be seen whether, over time and with prolonged remote learning, stress levels and depressive symptoms will change. The study would need to be repeated at several intervals. The survey was sent to all students at Opole University of Technology. In total, only 17.64% of the students completed it. We do not know the mental state of those who decided not to participate in the survey due to its voluntary nature. Only a study conducted on the entire available population (e.g., the entire year of a specific field of study) will provide full information about the scale of the problem. However, the study would no longer be voluntary, which is contrary to the requirements of the Bioethics Committee.

## Conclusion

Students during the COVID-19 pandemic and ongoing e-learning experience high levels of stress and depressive symptoms. The high percentage of students reporting suicidal thoughts is alarming. These results suggest that students require psychological support and appropriate assistance. Therefore, universities should implement intervention and educational programs, providing ad *hoc* assistance in the form of individual or group meetings with a psychologist (also in a remote form) and organizing workshops and webinars on strategies for managing stress. Students' mental health should be carefully monitored even after the pandemic is over, as the psychological consequences can linger much longer and affect many areas of life.

## Data Availability Statement

The raw data supporting the conclusions of this article will be made available by the authors, without undue reservation.

## Ethics Statement

The studies involving human participants were reviewed and approved by the Research Ethics Committee of the University of Physical Education in Wrocław, Poland (Ref. No. 1/2021). The patients/participants provided their written informed consent to participate in this study.

## Author Contributions

AR and JS-G: conceptualization. AR and AT: data curation. BC: formal analysis. AR: investigation, project administration. JS-G and AR: methodology. AR and BC: writing—original draft. AR, BC, and JS-G: writing—review editing. All authors contributed to the article and approved the submitted version.

## Conflict of Interest

The authors declare that the research was conducted in the absence of any commercial or financial relationships that could be construed as a potential conflict of interest.

## Publisher's Note

All claims expressed in this article are solely those of the authors and do not necessarily represent those of their affiliated organizations, or those of the publisher, the editors and the reviewers. Any product that may be evaluated in this article, or claim that may be made by its manufacturer, is not guaranteed or endorsed by the publisher.
