# Supplementary material for: Mental Health Conditions Among E-Learning Students During the COVID-19 Pandemic
Source: Front Public Health. 2022 May 17;10:871934. doi: 10.3389/fpubh.2022.871934 (PMC9152265; doi:10.3389/fpubh.2022.871934)
Supplement: Supplementary file 1 [file Table_1.DOCX]

Table S.1. Factor analysis of authors questionnaire

| Question | Factor 1 | Factor 2 |
| --- | --- | --- |
| E-learning isolated me from my friends. | 0.59 | 0.34 |
| E-learning has had a negative impact on my level of knowledge. | 0.88 | 0.07 |
| E-learning has had a negative impact on my level of practical skills. | 0.80 | 0.07 |
| E-learning reduced my motivation to learn. | 0.83 | 0.12 |
| I think I will get poorer grades on exams due to distance learning. | 0.73 | 0.19 |
| Due to pandemic, my skills in using modern technologies have improved. | 0.45 | -0.14 |
| E-learning forced to upgrade my computer (purchase of new hardware or software). | 0.06 | 0.80 |
| I use a PC which satisfies my needs. | 0.06 | 0.67 |
